# HLA class-I-peptide stability mediates CD8^+^ T cell immunodominance hierarchies and facilitates HLA-associated immune control of HIV

**DOI:** 10.1016/j.celrep.2021.109378

**Published:** 2021-07-13

**Authors:** Clarety Kaseke, Ryan J. Park, Nishant K. Singh, Dylan Koundakjian, Arman Bashirova, Wilfredo F. Garcia Beltran, Overbeck C. Takou Mbah, Jiaqi Ma, Fernando Senjobe, Jonathan M. Urbach, Anusha Nathan, Elizabeth J. Rossin, Rhoda Tano-Menka, Ashok Khatri, Alicja Piechocka-Trocha, Michael T. Waring, Michael E. Birnbaum, Brian M. Baker, Mary Carrington, Bruce D. Walker, Gaurav D. Gaiha

**Affiliations:** 1 Ragon Institute of MGH, MIT and Harvard, Cambridge, MA 02139, USA; 2 Harvard Radiation Oncology Program, Boston, MA 02114, USA; 3 Koch Institute for Integrative Cancer Research at MIT, Cambridge, MA 02142, USA; 4 Howard Hughes Medical Institute, Chevy Chase, MD 20815, USA; 5 Basic Science Program, Frederick National Laboratory for Cancer Research, Frederick, MD 21702, USA; 6 Department of Pathology, Massachusetts General Hospital, Boston, MA 02114, USA; 7 Department of Chemistry and Biochemistry, University of Notre Dame, Notre Dame, IN 46556, USA; 8 Harper Cancer Research Institute, University of Notre Dame, South Bend, IN 46556, USA; 9 Program in Virology, Harvard Medical School, Boston, MA 02114, USA; 10 Department of Ophthalmology, Massachusetts Eye and Ear, Boston, MA 02114, USA; 11 The Broad Institute, Cambridge, MA 02142, USA; 12 Massachusetts General Hospital Endocrine Unit and Department of Medicine, Harvard Medical School, Boston, MA 02114, USA; 13 Department of Biological Engineering, Massachusetts Institute of Technology, Cambridge, MA 02139, USA; 14 Center for the AIDS Programme of Research in South Africa, Durban 4001, South Africa; 15 Institute for Medical Engineering and Science and Department of Biology, Massachusetts Institute of Technology, Cambridge, MA 02139, USA; 16 Division of Gastroenterology, Massachusetts General Hospital, Boston, MA 02114, USA

**Keywords:** CD8+ T cells, immunodominance, HLA, HIV, epitopes, vaccine

## Abstract

Defining factors that govern CD8^+^ T cell immunodominance is critical for the rational design of vaccines for viral pathogens. Here, we assess the contribution of human leukocyte antigen (HLA) class-I-peptide stability for 186 optimal HIV epitopes across 18 HLA alleles using transporter associated with antigen processing (TAP)-deficient mono-allelic HLA-expressing cell lines. We find that immunodominant HIV epitopes increase surface stabilization of HLA class-I molecules in comparison to subdominant epitopes. HLA class-I-peptide stability is also strongly correlated with overall immunodominance hierarchies, particularly for epitopes from high-abundance proteins (e.g., Gag). Moreover, HLA alleles associated with HIV protection are preferentially stabilized by epitopes derived from topologically important viral regions at a greater frequency than neutral and risk alleles. These findings indicate that relative stabilization of HLA class-I is a key factor for CD8^+^ T cell epitope immunodominance hierarchies, with implications for HIV control and the design of T-cell-based vaccines.

## Highlights

• TAP-deficient HLA-expressing cells provide rapid assessment of HLA-peptide stability
• Immunodominant HIV CD8^+^ T cell epitopes are superior stabilizers of HLA molecules
• HLA class-I-peptide stability correlates with overall immunodominance hierarchies
• Protective HLA alleles are preferentially stabilized by highly networked epitopes

## Graphical abstract

**Figure:**
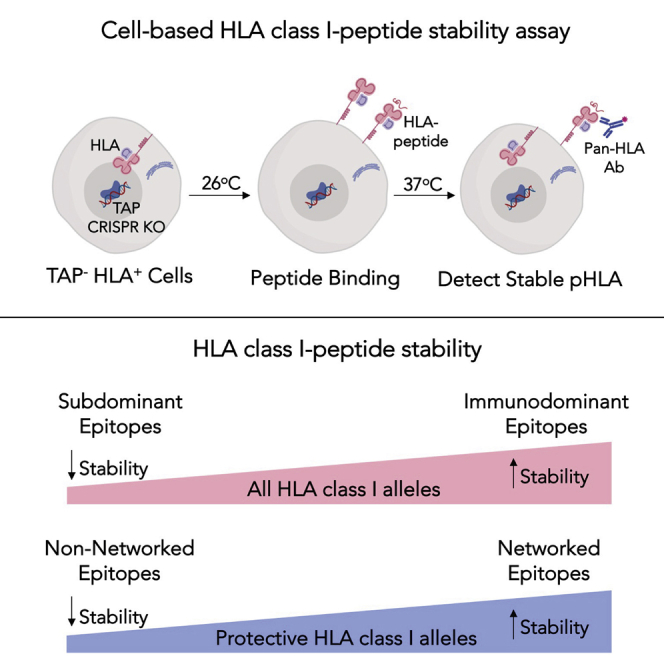

## Teaser

Kaseke et al. describe a cell-based HLA-peptide stability assay that leverages TAP-deficient mono-allelic HLA-expressing cell lines. Robust HLA stabilization is a common feature of immunodominant epitopes, and HLA-peptide stability correlates with immunodominance hierarchies. Epitopes derived from topologically constrained regions of the HIV proteome preferentially stabilize protective HLA class-I alleles.

## Introduction

CD8^+^ T cells play a key role in the suppression of viral infections through recognition of short viral peptides presented in complex with human leukocyte antigen (HLA) class-I glycoproteins (HLA-A, -B, and -C). While individual HLA class-I alleles can present thousands of unique peptides (Hunt et al., 2007; Vita et al., 2019), and viral genomes can encode many potential immunogenic sequences, in any given infection there is a remarkable restriction of CD8^+^ T cell responses to a limited set of pathogen-derived epitopes. This constraint on host cellular immunity, where some viral sequences are predominantly targeted over others, is known as immunodominance (ID) (Yewdell, 2006) and has important implications for the natural history of viral infections and the design of effective T-cell-based vaccines. However, the factors that govern CD8^+^ T cell ID are incompletely understood.

Numerous factors have been implicated in epitope ID patterns, such as proteasomal processing (Tenzer et al., 2009), cytosolic peptide stability (Lazaro et al., 2011), naive T cell precursor frequency (Kotturi et al., 2008), and utilization of public T cell receptor (TCR) sequences (Kløverpris et al., 2015; Lawson et al., 2001). However, previous work in mouse models has suggested that the ability of viral epitopes to bind and stabilize major histocompatibility complex (MHC) class-I and class-II molecules may also contribute to ID hierarchies (Busch and Pamer, 1998; Chen et al., 2000; Lazarski et al., 2005; Thirdborough et al., 2008; van der Burg et al., 1996). This is less well-established in humans, where HLA class-I-peptide stability has primarily been associated with epitope immunogenicity (Assarsson et al., 2007; Harndahl et al., 2012; Rasmussen et al., 2016). Thus, a comprehensive analysis of epitope-mediated stabilization of HLA class-I molecules, particularly for a broad set of HLA alleles and well-defined pathogens (e.g., human immunodeficiency virus [HIV]), would provide substantive insight into the contribution of surface HLA class-I-peptide stabilization to CD8^+^ T cell ID hierarchies.

Among the reasons why HLA class-I stability has not been more thoroughly evaluated is the labor and resource intensiveness of current techniques, such as thermal denaturation and circular dichroism of soluble HLA class-I-peptide complexes (Hellman et al., 2016; Morgan et al., 1997), which both require extensive HLA class-I protein expression, refolding, and purification. While high-throughput scintillation proximity assays to measure β2microglobulin (β2m) dissociation have been developed (Harndahl et al., 2012; Miles et al., 2011), they provide only indirect measures of HLA class-I-peptide stability.

To overcome these issues, we developed a direct cell-based HLA class-I-peptide stability assay by generating a panel of mono-allelic HLA class-I cell lines and subsequently editing the transporter associated with antigen processing (TAP) 1 gene using CRISPR/Cas9. TAP deficiency limits the transport of endogenous peptides to the endoplasmic reticulum to bind HLA class-I molecules, which in turn prevents their translocation to the cell surface as stable HLA-peptide complexes. However, when incubated at lower temperatures, TAP-deficient cells have been shown to accumulate peptide-receptive HLA class-I molecules at the surface (Day et al., 1995; Ljunggren et al., 1990), allowing them to be exogenously stabilized by delivered peptides of interest. The degree of stabilization can then be quantified by measurement of HLA class-I surface expression following incubation at 37°C, which promotes the endocytosis of unstable, non-peptide-bound MHC/HLA molecules (Montealegre et al., 2015). This has been previously performed for HLA-A^∗^02 using the TAP-deficient T2 lymphoblastoid cell line (Baas et al., 1992; Bell et al., 2009; Silva et al., 2020), but only rarely for other HLA alleles of global significance.

In this study, we developed and applied this assay for 18 HLA class-I alleles to demonstrate that relative HLA class-I-peptide stability is a key feature of ID CD8^+^ T cell epitopes and overall ID hierarchies, as defined by the frequency of CD8^+^ T cell epitope targeting in HIV-infected individuals (Streeck et al., 2009). In addition, we observed that protective HLA class-I alleles are better stabilized by epitopes derived from structurally constrained, topologically important regions of the HIV proteome, which mediate immune control of HIV when targeted by functional CD8^+^ T cells (Gaiha et al., 2019). Collectively, these data indicate that HLA class-I-peptide stability is an important factor to consider in defining immunogenic and protective CD8^+^ T cell epitopes for viral pathogens of global significance, with implications for the rational design of T cell-based vaccines.

## Results

### Cell-based HLA class-I-peptide stability assay

**Figure 1:**
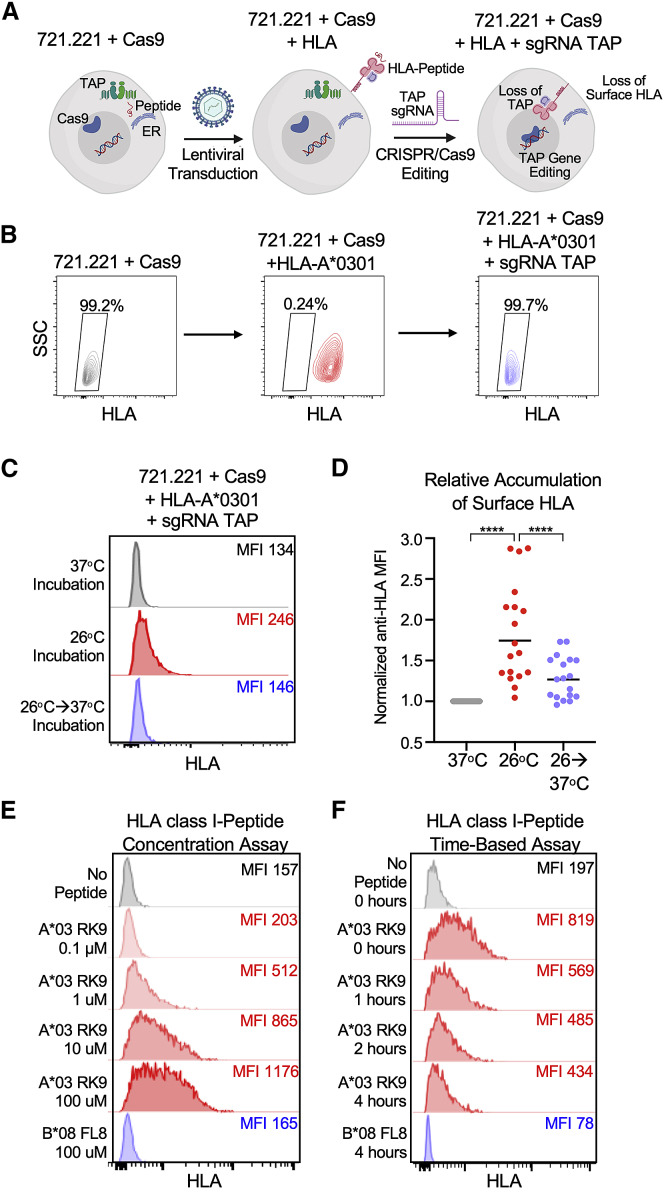
Cell-based HLA class-I-peptide stability assay (A) Schematic for generation of TAP-deficient mono-allelic HLA class-I-expressing 721.221 cell lines. Created with https://biorender.com. (B) Representative HLA class-I surface expression of 721.221 cells following transduction with Cas9-, HLA A^∗^0301-, and sgRNA TAP-expressing vectors with pan-HLA antibody W6/32. Flow plots presented following transduction with HLA-A^∗^0301, and sgRNA TAP are subclones. (C) Representative HLA class-I surface expression of TAP-deficient HLA-A^∗^0301 cells following overnight incubation at 37°C; 26°C; and after 2-h incubation at 37°C following overnight 26°C incubation. (D) Comparison of normalized anti-HLA MFI across all 18 TAP-deficient mono-allelic HLA class-I-expressing cell lines at the three indicated temperature conditions. Data are means of technical duplicates from an experiment performed twice. (E) Representative concentration-based stabilization of surface HLA-A^∗^0301 following incubation with no peptide, immunodominant (ID) HIV A^∗^0301 epitope RK9 (0.1-100 μM), and B^∗^08-restricted HIV epitope FL8. (F) Representative time-based stabilization of surface HLA-A^∗^0301 following overnight incubation at 26°C and with no peptide, RK9 peptide, or FL8 peptide, followed by incubation with BFA. Statistical comparisons were made using Mann-Whitney U test. For comparisons of more than two groups, Kruskal-Wallis test with Dunn’s pos hoc analyses were used. ^∗^p < 0.05; ^∗∗^p < 0.01; ^∗∗∗^p < 0.001; ^∗∗∗∗^p < 0.0001.

TAP-deficient mono-allelic HLA class-I-expressing cell lines were generated by sequential transduction of the HLA-null human B cell line 721.221 with lentiviral expression constructs encoding (1) Cas9 protein linked to blasticidin resistance gene via a self-cleaving P2A peptide (Sanjana et al., 2014); (2) 18 distinct HLA class-I alleles that provided >99% global coverage (Sette and Sidney, 1999; Sidney et al., 2008) (*A^∗^0101*, *A^∗^0201*, *A^∗^0301*, *A^∗^2402*, *B^∗^0702*, *B^∗^0801*, *B^∗^1402*, *B^∗^1501*, *B^∗^2705*, *B^∗^3501*, *B^∗^3901*, *B^∗^4001*, *B^∗^4402*, *B^∗^5201*, *B^∗^5701*, *B^∗^5801*, *B^∗^8101*, and *Cw^∗^0701*) under puromycin resistance linked via an internal ribosome entry sequence (IRES); and (3) a single guide RNA (sgRNA) directed toward exon 3 of the human *TAP1* under neomycin/G418 resistance linked via an IRES (Figure 1A). The 721.221-Cas9 cells were subcloned following HLA class-I gene transduction to yield >99% HLA-positive cell populations, as shown for HLA-A^∗^0301 (Figure 1B). CRISPR/Cas9 editing of the TAP1 gene, which was confirmed by amplicon sequencing of genomic DNA for each mono-allelic cell line, yielded a range of negative HLA surface expression (8.7%–85.5%; Figure S1A). These cell lines were further subcloned to yield stable populations in which less than <2.5% showed evidence of surface HLA expression (Figures 1B and S1B).

Given that TAP-deficient cells have been shown to accumulate peptide-receptive HLA class-I molecules at the surface when incubated at lower temperatures (Day et al., 1995; Ljunggren et al., 1990; Montealegre et al., 2015), we first evaluated whether the CRISPR/Cas9-edited TAP-deficient HLA class-I cell lines would also accumulate HLA class-I molecules at the cell surface. We therefore incubated each mono-allelic cell line at 26°C for 18 h and subsequently measured surface HLA class-I surface expression using the pan-HLA antibody W6/32 (Parham et al., 1979). At 26°C, surface HLA expression was readily detectable for the panel of 18 mono-allelic HLA cell lines but decreased significantly after 2 h incubation at 37°C (Figures 1C and 1D). This demonstrated that HLA molecules were successfully accumulating at the cell surface when incubated at 26°C and then downmodulated from the surface following incubation at 37°C, indicating that these TAP-deficient cell lines could be appropriately utilized for assessments of HLA class-I-peptide stability.

In the stability assay, TAP-deficient cells were incubated for 18 h at 26°C with 8–11 amino acid peptides prior to a 2 h incubation at 37°C. Stable HLA class-I-peptide complexes were then detected on the cell surface by anti-HLA antibody, and the change in anti-HLA mean fluorescence intensity (MFI) from baseline indicates the degree of HLA molecule stabilization by the tested peptide. We also found that the addition of soluble β2m significantly enhanced the surface stabilization of HLA class-I molecules by peptides without significantly affecting baseline levels of HLA expression (Figure S2). As a representative example of the assay, we incubated TAP-deficient HLA-A^∗^0301 mono-allelic cells with the well-established ID HIV A^∗^03-restricted RK9 epitope (RLRPGGKKK, Gag p17 20-28) (Streeck et al., 2009) at increasing peptide concentrations (0.1–100 μM) and detected a concentration-dependent increase in surface HLA-A^∗^0301 expression (Figure 1E). Importantly, we observed no increase in surface expression following incubation with the irrelevant HIV B^∗^08-restricted FL8 epitope (FLKEKGGL, Nef 90-97). We also performed a time-dependent assay with Brefeldin A (BFA), which prevents the transport of newly synthesized HLA class-I molecules to the cell surface and thereby allows for an assessment of the stability of existing surface HLA class-I-peptide complexes (Montealegre et al., 2015). TAP-deficient HLA-A^∗^0301 cells were incubated with HIV RK9 peptide (10 μM) for 18 h at 26°C prior to incubation at 37°C in the presence of BFA. Measurement of HLA class-I surface expression by flow cytometry revealed a time-dependent decrease in HLA-A^∗^0301 surface expression but the presence of ∼40% stable HLA-A^∗^0301-RK9 peptide complexes after 4 h (Figure 1F). This was in contrast to the negligible surface expression at 4 h following incubation with the irrelevant HIV B^∗^08-restricted FL8 epitope. Collectively, these results indicated that our CRISPR/Cas9-edited TAP-deficient HLA class-I cell lines were well suited for comprehensive HLA class-I-peptide stability evaluation by both concentration- and time-dependent assays.

### Surface stabilization of HLA class-I molecules by ID and SubD HIV CD8+ T cell epitopes

**Figure 2:**
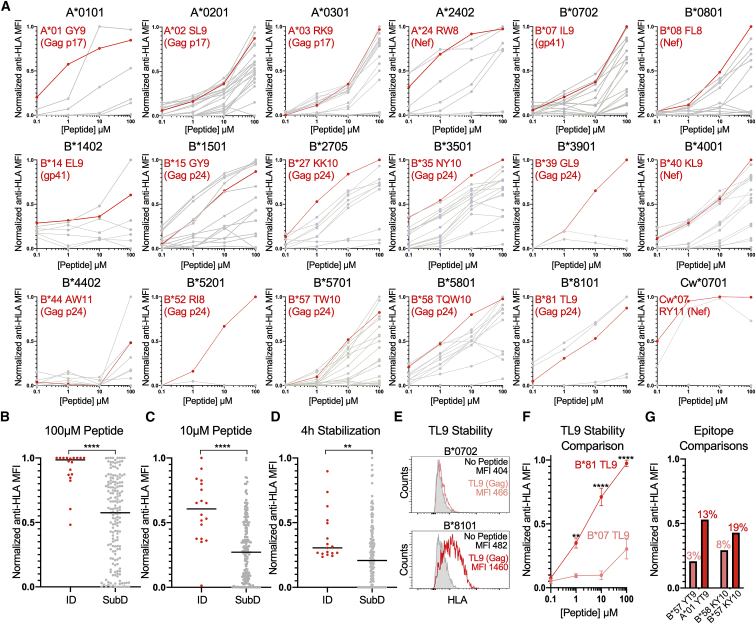
HLA class-I-peptide stability is a key feature of ID HIV CD8+ T cell epitopes (A) Concentration-based HLA class-I stabilization of 186 known optimal HIV CD8^+^ T cell epitopes (0.1–100 μM) across 18 TAP-deficient mono-allelic HLA class-I-expressing cell lines. The y axis depicts the anti-HLA MFI normalized to the highest value for each HLA class-I allele (0–1). Known ID HIV epitopes based on frequency of CD8^+^ T cell targeting are indicated in red. Subdominant (SubD) and no-peptide controls are presented in gray. Each data point is a mean of technical duplicates from an experiment performed twice. (B–D) Comparison of normalized anti-HLA MFI of ID and SubD epitopes at 100-μM and 10-μM peptide concentrations, and following 4 h incubation with BFA, respectively. (E) HLA class-I-peptide stabilization of TAP-deficient B^∗^0702- and B^∗^8101-expressing cell lines following incubation with TL9 peptide (10 μM). (F) Comparison of normalized anti-HLA MFI for B^∗^0702 and B^∗^8101 following incubation with TL9 peptide (0.1–100 μM). Each data point is a mean ± SEM of technical duplicates from an experiment performed three times. (G) Comparison of normalized anti-HLA MFI for HLA-A^∗^0101- and HLA-B^∗^5701-expressing cell lines following incubation with YT9 peptide (10 μM) and HLA-B^∗^5701- and HLA-B^∗^5801-expressing cell lines following incubation with KY10 peptide (10 μM). Data are means of technical duplicates from an experiment performed twice. Frequency of CD8+ T cell targeting, as previously determined (Streeck et al., 2009), is indicated above each anti-HLA MFI value. Statistical comparisons were made using Mann-Whitney U test. ^∗^p < 0.05; ^∗∗^p < 0.01; ^∗∗∗^p < 0.001; ^∗∗∗∗^p < 0.0001.

To assess the impact of concentration- and time-dependent HLA class-I-peptide stability on CTL epitope ID, we tested 186 HIV epitopes from the optimal “A list” that were restricted by the 18 HLA class-I alleles present in the panel of TAP-deficient mono-allelic HLA-expressing cell lines (Table S1) (Llano et al., 2019). As demonstrated by the concentration-dependent assay, we observed a range of surface HLA class-I stabilization by the tested peptides across all HLA alleles (Figure 2A). Notably, the known ID HIV epitope for each HLA class-I allele, as determined by the frequency of CD8^+^ T cell epitope targeting among 527 individuals during acute infection (Streeck et al., 2009), was consistently among the highest HLA class-I stabilizers. Comparative analysis between ID and subdominant (SubD) epitopes at 100 μM and 10 μM revealed a highly statistically significant difference in normalized anti-HLA MFI (Figures 2B and 2C). This was also observed in the time-dependent assay (Figure S3), in which the level of surface HLA class-I expression after 4 h incubation in the presence of BFA differentiated ID from SubD epitopes (Figure 2D).

To normalize for factors that may contribute to CD8^+^ T cell epitope ID, we focused on the HIV Gag TL9 epitope (Gag p24 48-56; TPQDLNTML), which is presented by a number of alleles within the HLA-B7 supertype but with markedly different patterns of ID. For example, while this epitope is ID for B^∗^8101 and is targeted by >70% of B^∗^8101 individuals, it is rarely targeted by individuals who express B^∗^0702 (∼5%) (Leslie et al., 2006). We therefore compared the concentration-dependent stabilization of B^∗^0702 and B^∗^8101 by TL9 and found a highly statistically significant difference, with B^∗^8101 being stabilized at substantially higher surface levels in comparison to B^∗^0702 across a range of TL9 peptide concentrations (Figures 2E and 2F). We observed similar differences in HLA class-I-peptide stability for additional promiscuous HIV epitopes with distinct levels of epitope targeting by CD8^+^ T cells, such as YT9 (Nef 120-128; YFPDWQNYT; A^∗^01, B^∗^57) and KY10 (Rev 14-23; KAVRLIKFLY; B^∗^57, B^∗^58) (Figure 2G). Collectively, these results suggested that relative HLA class-I-peptide stability is a key component of CD8^+^ T cell epitope ID in natural HIV infection.

To confirm these observations and further validate the cell-based stability assay, we used an orthogonal approach to evaluate the relationship between HLA class-I-peptide stability and ID by assessing thermal denaturation of soluble HLA class-I-peptide complexes (Hellman et al., 2016). In this assay, soluble HLA class-I monomers with bound peptide are incubated in the presence of an environmentally sensitive fluorescent molecule whose fluorescence is enhanced when bound to exposed hydrophobic surfaces during protein unfolding. As a result, the thermal stability (T_m_, defined as the temperature at which 50% of the protein is unfolded) of an HLA class-I-peptide complex can be determined by fluorometric measurements during incremental temperature increases, which serve as a proxy for the stabilizing capacity of a bound epitope (Hellman et al., 2016).

**Figure 3:**
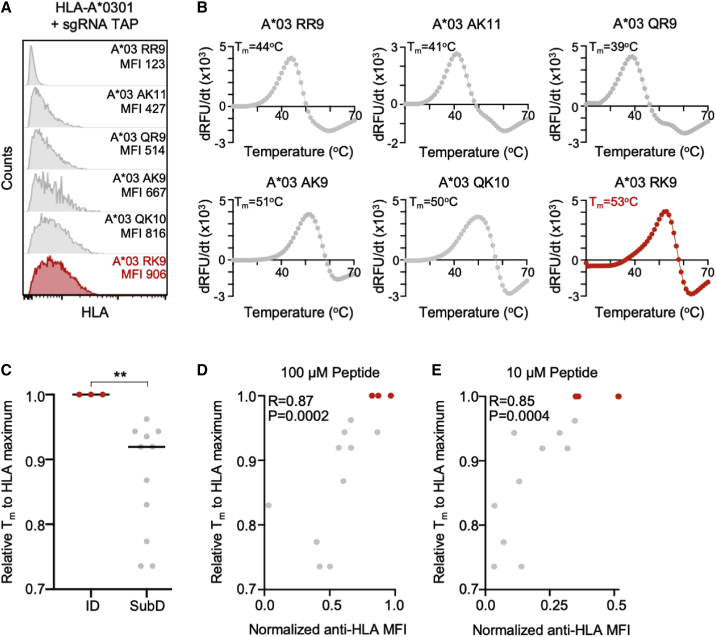
Cell-based HLA class-I-peptide stability strongly correlates with thermal denaturation (A) HLA-A^∗^0301 surface stabilization following incubation with indicated HIV epitopes. ID A^∗^0301 RK9 epitope is shown in red. (B) Representative thermal denaturation of HLA-A^∗^0301-peptide monomers for indicated HIV epitopes. The x axis depicts temperature (20°C–70°C). The y axis depicts the derivative of the temperature versus fluorescence (–dRFU/dT). The thermal stability (Tm) is indicated for each HLA-A^∗^0301-peptide complex. (C) Comparison of relative thermal stability temperature (T_m_), normalized to the maximum T_m_ for each HLA allele (0–1), for ID and SubD epitopes. Each data point is a mean of technical triplicates from an experiment performed twice. (D and E) Scatterplot of normalized anti-HLA MFI (x axis) at 100-μM and 10-μM peptide concentration with relative T_m_ to HLA maximum (y axis). Correlations were calculated by Spearman’s rank correlation coefficient. Statistical comparisons were made using Mann-Whitney U test. ^∗^p < 0.05; ^∗∗^p < 0.01; ^∗∗∗^p < 0.001; ^∗∗∗∗^p < 0.0001.

We therefore expressed and refolded soluble monomers for three HLA class-I alleles (HLA-A^∗^0201, HLA-A^∗^0301, and HLA-B^∗^5701) in complex with ID (n = 3) and SubD HIV epitopes (n = 10), prior to thermal denaturation. As demonstrated for HLA-A^∗^0301, which had a broad range of cell-based HLA class-I stabilization with distinct peptide epitopes (Figure 3A), the T_m_ of the ID RK9 epitope was elevated (53°C) in comparison to T_m_s of several SubD HLA-A^∗^0301 epitopes (39°C–51°C) (Figure 3B). This relative difference in T_m_ between ID and SubD epitopes was also observed for HLA-A^∗^02 and HLA-B^∗^57 (Figure S4) and collectively was statistically significant (Figure 3C). Comparison of relative T_m_ of soluble HLA monomers with normalized anti-HLA MFI values generated by the cell-based assay revealed a striking and highly significant correlation at peptide concentrations of 100 μM (R = 0.87, p = 0.0002) and 10 μM (R = 0.85, p = 0.0004) (Figures 3D and 3E). This demonstrated that the cell-based HLA class-I-peptide stability assay was comparable to the thermal denaturation assay and further confirmed the role of relative HLA class-I-peptide stability in defining ID epitopes.

### Correlation of HLA class-I-peptide stability with CD8^+^ T cell epitope ID hierarchies in acute HIV infection

**Figure 4:**
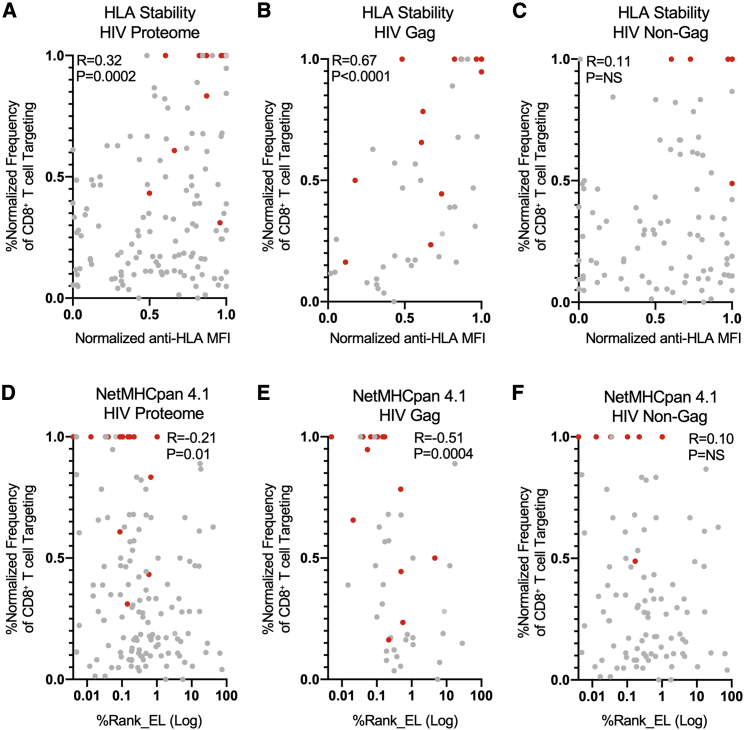
HLA class-I-peptide stability is positively correlated with frequency of CD8+ T cell epitope targeting and outperforms affinity-based predictions (A–C) Scatterplots of normalized anti-HLA MFI (x axis) with normalized frequency of CD8+ T cell epitope targeting (y axis) during acute infection for the whole-HIV proteome, Gag-derived epitopes, and non-Gag-derived epitopes from 527 HIV-infected individuals (Streeck et al., 2009). (D–F) Scatterplots of NetMHCpan 4.1%Rank_EL (x axis) with normalized frequency of CD8+ T cell epitope targeting (y axis) during acute infection for the whole-HIV proteome epitopes, Gag-derived epitopes, and non-Gag-derived epitopes. ID HIV epitopes during acute HIV infection are shown in red. Correlations were calculated by Spearman’s rank correlation coefficient.

Given the link between relative HLA class-I-peptide stability and ID HIV CD8^+^ T cell epitopes, we next sought to evaluate its contribution to overall ID hierarchies. To accomplish this, we utilized a published dataset that determined the frequency of CD8^+^ T cell epitope targeting in 527 HIV-infected individuals during acute HIV infection (Streeck et al., 2009) and normalized these targeting values within each HLA allele. Correlation of HLA class-I-peptide stability for each epitope with its normalized frequency of CD8^+^ T cell targeting frequency during acute HIV infection was positively correlated (R = 0.32) and highly statistically significant (p = 0.0002) (Figure 4A), indicating the association between HLA stabilizing capacity and epitope ID hierarchies.

We next investigated the additional contribution of viral protein abundance to ID hierarchies, given previous studies of cancer neoantigens that demonstrated a link between protein and mRNA transcript levels with antigen presentation (Abelin et al., 2017; Bassani-Sternberg et al., 2015). Since the Gag protein is expressed in infected cells at levels >1 log higher than other viral proteins (Ambrose and Aiken, 2014; Lee et al., 2012; Zhu et al., 2003), we separated epitopes into those derived from Gag and non-Gag sources. Correlation of HLA class-I-peptide stability with CD8^+^ T cell targeting during acute infection revealed a robust and highly significant correlation for Gag-derived epitopes (R = 0.67, p < 0.0001) (Figure 4B), but not for epitopes from non-Gag proteins (Figure 4C). This revealed that the combination of HLA class-I-peptide stability and viral protein abundance is likely a major contributor to CD8^+^ T cell epitope ID hierarchies during acute HIV infection.

In order to assess whether HLA class-I-peptide stability provides insight beyond standard binding affinity, we used the NetMHCpan 4.1 server (http://www.cbs.dtu.dk/services/NetMHCpan/) to obtain HLA-peptide affinity values for each HIV epitope (Table S1). While predicted affinity values and measures of HLA class-I-peptide affinity were significantly correlated (Figure S5), the magnitude of Spearman correlation coefficients for HLA-peptide affinity and the normalized frequency of CD8^+^ T cell epitope targeting were notably lower in comparison to HLA class-I-peptide stability for both whole-HIV proteome and Gag epitopes (R = 0.21 versus 0.31, HIV proteome; R = 0.51 versus 0.67, Gag) (Figures 4D and 4E). Comparison of HLA-peptide affinity with CD8^+^ T cell targeting for non-Gag epitopes was not significantly correlated, further highlighting the role of viral protein abundance in delineating ID hierarchies (Figure 4F). Collectively, these findings indicate that an epitope’s ability to bind and stabilize HLA class-I molecules is a better overall predictor of CD8+ T cell ID hierarchies than HLA binding affinity alone, further supporting the value of determining the HLA stabilizing capacity of candidate epitopes to inform the rational design of T-cell-based vaccines for HIV and other pathogens.

### Surface stabilization of protective and non-protective HLA class-I alleles by topologically important epitopes

Recent work from our laboratory demonstrated that functional CD8^+^ T cell targeting of epitopes derived from structurally constrained, topologically important regions of the viral proteome (“highly networked” epitopes; Table S1) is associated with successful immune control of HIV (Gaiha et al., 2019). In addition, we found that epitopes presented by protective HLA class-I alleles (*B^∗^1402*, *B^∗^2705*, *B^∗^5201*, *B^∗^5701*, *B^∗^5801*, and *B^∗^8101*) (Pereyra et al., 2010; Lazaryan et al., 2006; Ntale et al., 2012; O’Brien et al., 2001) were more often derived from highly networked viral regions in comparison to neutral and risk alleles. The data suggested that the increased likelihood of presenting a highly networked epitope may account for the enrichment of protective HLA class-I alleles within HIV controllers. However, we had not considered whether protective, neutral, and risk HLA class-I alleles were differentially stabilized by highly networked epitopes (epitope network score > 3.06) or non-networked epitopes (Gaiha et al., 2019), which would provide a further biochemical explanation for their distinct phenotypes.

**Figure 5:**
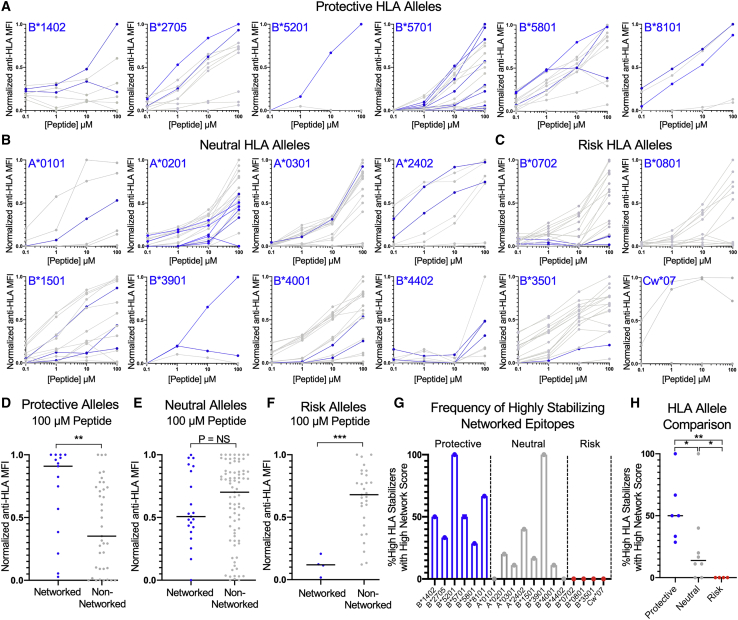
Protective HLA class-I alleles are preferentially stabilized by topologically important epitopes (A–C) Concentration-based HLA class-I stabilization of optimal HIV CD8+ T cell epitopes (0.1–100 μM) across TAP-deficient mono-allelic HLA class-I-expressing cell lines for protective, neutral, and risk HLA class-I alleles, respectively. The y axis depicts the anti-HLA MFI normalized to the highest value for each HLA class-I allele (0–1). Highly networked CD8+ T cell epitopes, as previously defined (Gaiha et al., 2019), are indicated in blue. Each data point is a mean of technical duplicates from an experiment performed twice. (D–F) Comparison of normalized anti-HLA MFI of networked and non-networked epitopes for protective, neutral, and risk HLA class-I alleles, respectively. (G) Percentage of highly stabilizing HLA class-I epitopes that are highly networked for each protective (blue), neutral (gray), and risk (red) allele. (H) Comparison of percentage of highly networked, highly stabilizing HLA class-I epitopes across protective, neutral, and risk HLA class-I alleles. Statistical comparisons were made using Mann-Whitney U test. For comparisons of more than two groups, Kruskal-Wallis test with Dunn’s pos hoc analyses were used. ^∗^p < 0.05; ^∗∗^p < 0.01; ^∗∗∗^p < 0.001; ^∗∗∗∗^p < 0.0001.

We therefore evaluated the relative HLA class-I-peptide stability of all epitopes across the panel of 18 TAP-deficient mono-allelic HLA class-I-expressing cell lines and observed that highly networked epitopes were among the best stabilizers for protective HLA class-I alleles (Figure 5A) but were less likely to be among the top stabilizers for neutral and risk alleles (Figures 5B and 5C). Comprehensive analysis of all epitopes revealed that protective HLA class-I alleles had significantly higher levels of surface HLA stabilization by highly networked epitopes in comparison to non-networked epitopes (Figure 5D). In contrast, neutral alleles were not preferentially stabilized by highly networked epitopes (Figure 5E), and risk alleles were preferentially stabilized by non-networked epitopes in a statistically significant manner (Figure 5F). Assessment of the frequency of highly networked epitopes within peptides that achieved high HLA class-I stabilization for each allele (defined as >50% relative stabilization to the best stabilizer at 100 μM) further demonstrated the propensity of protective HLA class-I alleles to present highly networked epitopes, in contrast to neutral and risk alleles (Figures 5G and 5H). Within the neutral alleles, HLA-B^∗^3901 appeared to be better stabilized by highly networked epitopes, which may explain its previous association with lower viral loads (Leslie et al., 2006; Ramírez de Arellano et al., 2019), while HLA-A^∗^2402 was also stabilized by a highly networked epitope, although it was derived from a lower-abundance protein (Nef). Collectively, this suggests that the variable disease progression of protective, neutral, and risk HLA class-I alleles may be the result of their differential capacity to be stabilized by highly networked epitopes and, therefore, the ability of CD8^+^ T cells to consistently target the virus at structurally constrained, topologically important regions.

## Discussion

Delineating the factors that govern CD8^+^ T cell epitope ID has important implications for the development of rational T-cell-based vaccines for viral pathogens. Toward this objective, we comprehensively assessed the relative HLA class-I-peptide stability for 186 optimal HIV epitopes across 18 globally relevant HLA alleles and demonstrate that HLA class-I-peptide stability and protein abundance are key features of ID epitopes and overall ID hierarchies during acute HIV infection. We also show that protective HLA class-I alleles are better stabilized by epitopes derived from structurally constrained, topologically important regions of the viral proteome, providing a further biochemical basis for why these HLA alleles may be consistently enriched among individuals who spontaneously control HIV.

The resource-intensive nature of current approaches to assess HLA class-I-peptide stability, such as thermal denaturation or measurement of dissociation of soluble HLA class-I-peptide complexes, has limited their application to broad assessments of ID hierarchies (Hellman et al., 2016; Morgan et al., 1997). In addition, higher-throughput β2m dissociation methods still require HLA protein expression and provide only an indirect assessment of the interaction between peptides and HLA class-I molecules (Harndahl et al., 2012). In contrast, the cell-based HLA class-I-peptide stability assay described in this report provides a direct measure of peptide-HLA class-I interactions and is far less resource intensive, relying solely on peptide synthesis, rather than on the generation of complete HLA class-I-peptide protein complexes. Moreover, the utilization of flow cytometry allows for a rapid and sensitive assessment of peptide stabilizing capacity, while still yielding relative HLA class-I-peptide stability values that are highly correlated to thermal denaturation. While the cell-based assay does not specifically take into account proteasomal processing due to the engineered TAP deficiency, this has been shown to play a less dominant role in delineating immunogenic epitopes due to the promiscuous activity of the immunoproteasome (Abelin et al., 2017). In addition, this limitation would not readily apply to a well-studied pathogen such as HIV, for which epitopes have been comprehensively defined.

We therefore assessed the relative HLA class-I-peptide stability of optimal HIV CD8^+^ T cell epitopes, which distinguished ID CD8^+^ T cell epitopes from SubD epitopes. For overall CD8^+^ T cell ID hierarchies, we also observed a positive correlation with HLA class-I-peptide stability, which substantially improved when evaluated in the context of epitopes derived from high-abundance HIV Gag proteins. This is consistent with previous mass spectrometry profiling studies and comprehensive assessments of tumor epitope immunogenicity that also observed an association between protein abundance and HLA class-I-peptide presentation (Abelin et al., 2017; Bassani-Sternberg et al., 2015; Wells et al., 2020). Interestingly, we observed weaker correlations when predicted HLA binding affinities from NetMHCpan 4.1 were used, suggesting that the combination of binding and stabilization of HLA class-I alleles provides additional value beyond binding affinity alone in the determination of ID hierarchies. This is consistent with prior studies that utilized an affinity-balanced approach and demonstrated that HLA class-I-peptide stability was more significantly associated with epitope immunogenicity than affinity (Harndahl et al., 2012; Rasmussen et al., 2016; van der Burg et al., 1996).

With respect to the development of a protective T-cell-based vaccine, we recently demonstrated that individuals who successfully control HIV in the absence of therapy preferentially target CD8^+^ T cell epitopes derived from structurally constrained, topologically important regions of the viral proteome using an approach known as structure-based network analysis (Gaiha et al., 2019). While this was observed irrespective of an individual’s HLA haplotype, we also found that CD8^+^ T cell epitopes presented by protective HLA class-I alleles (Pereyra et al., 2010) were more likely to be derived from highly networked regions of the viral proteome in comparison to neutral and risk alleles. This observation was even more pronounced when the analysis was limited to ID epitopes (Gaiha et al., 2019), and therefore is consistent with highly networked epitopes being among the best stabilizers of protective HLA class-I alleles. This was in stark contrast to neutral and risk alleles, which were both either similarly stabilized or preferentially stabilized by non-networked epitopes. These findings thereby provide a putative biochemical explanation as to why certain HLA class-I alleles, such as HLA-B^∗^57 and B^∗^27, are consistently enriched within cohorts of HIV controllers. For neutral alleles that were stabilized by highly networked epitopes (e.g., A^∗^2402, B^∗^3901), these epitopes were either derived from lower abundance proteins, which are less frequently targeted by CD8^+^ T cells, or the allele (B^∗^3901) had previously been associated with lower viral loads (Leslie et al., 2006; Ramírez de Arellano et al., 2019). Collectively, these observations indicate that vaccine immunogens comprising highly networked epitopes may be further optimized by excluding highly stabilizing, poorly networked epitopes, particularly from high-abundance proteins.

In addition to HIV, the findings presented here are broadly applicable to the design of T-cell-based vaccines for a wide range of globally important pathogens. Specifically, the data suggest that CD8^+^ T cell epitopes should be selected based on their ability to stabilize HLA class-I molecules and their derivation from high-abundance viral proteins. The generation of 18 TAP-deficient mono-allelic HLA class-I cell lines allowed the cell-based stability assay to identify epitopes that provide coverage of >99% of the global population (Sette and Sidney, 1999; Sidney et al., 2008). Thus, our results elucidate an important role for relative HLA class-I-peptide stability in the delineation of CD8^+^ T cell ID hierarchies during HIV infection, with additional implications for HLA-associated immune control of HIV. We envision that the cell-based HLA class-I stability assay we describe in this report will also allow for the rapid identification of highly stabilizing, immunogenic CD8^+^ T cell epitopes for a diverse array of pathogens of global relevance.

### Limitations of study

In the present study, it should be noted that our comparisons of HLA class-I-peptide stability to HLA-peptide affinity utilized predicted affinity values from NetMHCPan 4.1. While several of the HIV epitopes that were evaluated in this study were part of the NetMHCPan 4.1 training set—and therefore incorporated measured binding affinities—this was not comprehensive for all epitopes. In addition, we were only able to evaluate three HLA alleles and 14 HLA-peptide complexes by soluble thermal denaturation. While T_m_ values and cell-based HLA class-I-peptide stability were highly correlated, future studies evaluating additional HLA alleles and HLA-peptide complexes will help further confirm the role of HLA class-I-peptide stability in mediating ID hierarchies. Lastly, CD8^+^ T cell targeting data (Streeck et al., 2009) were available for only 74.2% of the epitopes analyzed by HLA class-I-peptide stability assay. Additional targeting data would further refine correlations between HLA-peptide stability and ID.

## STAR★Methods

### Key resources table

**Table:** 

REAGENT or RESOURCE	SOURCE	IDENTIFIER
**Antibodies**		

Mouse monoclonal Anti-HLA ABC (clone W6/32) labeled with APC fluorophore	Biolegend	Cat# 311410; RRID:AB_314879
LIVE/DEAD Violet Viability	Life Technologies	Cat# L34960

**Chemicals, peptides, and recombinant proteins**		

HIV Epitope Peptides	MGH Peptide Core	N/A
β2-Microglobulin	Sino Biological	Cat# 11976-H08H
Soluble HLA class I monomers	This paper	N/A

**Deposited data**		

CRISPR amplicon sequencing of human TAP1 gene of CRISPR/Cas9-edited mono-allelic HLA class I-expresssing cell lines	Mendeley Data	https://doi.org/10.17632/hh46ymyxkk.1

**Experimental models: Cell lines**		

Human: 721.221 cells	A gift from Bruce Walker, Ragon Institute	N/A
Human: HEK293T cells	ATCC	CRK-1573
Human: 721.221 cells + Cas9 + HLA + sgRNA TAP	This paper	N/A

**Oligonucleotides**		

Primer: TAP gDNA Sequencing Forward: AGTCTGTTCCCTGAACACAC	This paper	N/A
Primer: TAP gDNA Sequencing Reverse: GGAGATCAAAGCAGATGTATG	This paper	N/A
TAP sgRNA: CACCGCGGGATCTATAACAACACCA	McKinley et al., 2015	TAP sgRNA #5

**Recombinant DNA**		

LentiCas9-Blast9	Sanjana et al., 2014	Addgene Plasmid #52692
psPAX2	A gift of Didier Trono, EPFL	Addgene Plasmid #12260
pLenti-sgRNA	A gift of Eric Lander/David Sabatini, Broad Institute/Whitehead Institute	Addgene Plasmid #71409
pLenti-TAP sgRNA	This paper	N/A
pHEF-VSVG	Coleman et al., 2003	Addgene Plasmid #22501
pLVX-EF1α-IRES-Puro	Clontech	Cat# 631988
pLVX-EF1α-IRES-Puro with HLA inserts	This paper	N/A
pET28a with HLA construct and β2M	Pierce et al., 2014	N/A

### Resource availability

#### Lead contact

Further information and requests for resources and reagents should be directed to and will be fulfilled by the lead contact, Gaurav D. Gaiha (ggaiha@mgh.harvard.edu)

#### Materials availability

All requests for resources and reagents should be directed to and will be fulfilled by the lead contact author. All reagents will be made available on request after completion of a Materials Transfer Agreement.

#### Data and code availability

All data supporting the findings of this study available within the paper and are available from the corresponding author upon request. Sequence data of CRISPR amplicons of the TAP1 gene is available at Mendeley Data (https://doi.org/10.17632/hh46ymyxkk.1).

### Experimental models and subject details

#### Cell lines

The human female B cell line 721.221 were generated previously by γ-radiation of 721 cells and do not express HLA A and B alleles (Shimizu and DeMars, 1989). These cell lines were maintained in RPMI-1640 medium (Sigma-Aldrich) supplemented with 10% (v/v) FBS (Sigma-Aldrich) and 1X Penicillin-Streptomycin-L-Glutamine mixture (GIBCO). HEK293T cells used for lentivirus production were maintained in advanced DMEM (Sigma-Aldrich) supplemented with 10% FBS, 2mM L-glutamine (GIBCO), 1X non-essential amino acids (GIBCO) and 1X sodium pyruvate (GIBCO).

#### TAP-deficient mono-allelic HLA class I-expressing cell line generation

Lentiviral transduction for each sequential transduction was performed as described (Garcia-Beltran et al., 2016). 721.221 cells were transduced with lentivirus encoding LentiCas9-Blast and then selected with 5 μg/mL blasticidin (Invivogen). 721.221 + Cas9 cells were subsequently transduced with lentivirus encoding HLA class I genes and selected in 0.5 μg/ml puromycin (Invivogen). High HLA class I expressing cells were subcloned by limiting dilution. High-expressing HLA class I clones were then transduced with lentivirus encoding pLenti-sgRNA targeting exon 3 of the human TAP1 gene, followed by selection in 1.5 mg/ml G418 (Invivogen). Cells with low HLA class I surface expression following were subcloned by limiting dilution. Cas9, HLA and TAP sgRNA-expressing 721.221 cells were maintained in 5 μg/mL blasticidin (Invivogen), 0.5 μg/ml puromycin (Invivogen) and 1.5 mg/ml G418 (Invivogen).

### Method details

#### Recombinant DNA constructs

LentiCas9-Blast was a gift from Feng Zhang (Addgene plasmid # 52962; http://addgene.org/52962; RRID:Addgene_52962). The plasmid psPAX2 was a gift from Didier Trono (Addgene plasmid # 12260; htto://addgene.org/12260; RRID:Addgene_12260). The plasmid pLenti-sgRNA was a gift from Eric Lander and David Sabatini (Addgene plasmid # 71409; http://addgene.org/71409; RRID:Addgene_71409). The plasmid pHEF-VSVG was a gift from Sergey Kasparov (Addgene plasmid # 22501; http://addgene.org/22501; RRID:Addgene_22501). The HLA expression set included *A^∗^0101, A^∗^0201, A^∗^0301, A^∗^2402, B^∗^0702, B^∗^0801, B^∗^1402, B^∗^1501, B^∗^2705, B^∗^3501, B^∗^3901, B^∗^4001, B^∗^4402, B^∗^5201, B^∗^5701, B^∗^5801, B^∗^8101* and *Cw^∗^0701*. Synthetic HLA allele fragments (LifeSct) were cloned into a modified pLVX-EF1α-IRES-Puro (Clontech) vector (*46*), in which EF1α was replaced with the SFFV promoter (pLVX-SFFV-IRES-Puro). This expression cassette also encoded ZsGreen linked via self-cleaved P2A peptide to HLA with a FLAG-tag at its N terminus. These elements were removed by enzymatic digestion with EcoRI (NEB) and NotI (NEB) prior to re-cloning of HLA fragments. TAP sgRNA construct (5′-CACCGCGGGATCTATAACAACACCA-3′) was cloned into pLenti-sgRNA (McKinley et al., 2015; Park et al., 2017). All plasmids were confirmed by complete plasmid sequencing (MGH DNA Core).

#### CRISPR amplicon sequencing of human TAP1 gene of CRISPR/Cas9-edited mono-allelic HLA class I-expressing cell lines

To confirm successful editing of the human TAP1 gene, genomic DNA was isolated from all 18 CRISPR/Cas9 edited mono-allelic HLA class I cell lines using the Qiaamp DNA mini kit (QIAGEN), according to the manufacturer’s instructions. CRISPR amplicons were generated by PCR amplification using 2 μg of genomic DNA and Q5 High-Fidelity 2X Master Mix (NEB) at an in an Eppendorf Mastercycler Pro (98°C for 30 s, 25 cycles of 98°C for 30 s, 51°C for 30 s, 72°C for 30 s, final extension at 72°C for 2 min). Primers for *TAP1* flanked the sgRNA target (5′-AGTCTGTTCCCTGAACACAC-3′, 5′-GGAGATCAAAGCAGATGTATG-3′) to selectively generate a 252 bp DNA amplicon. Amplicon sequencing was carried out by the MGH DNA Sequencing Core Facility.

#### Analysis of CRISPR sequencing data

Analysis of sequenced PCR-amplified CRISPR guide target regions was animated using the Perl (Wall et al., 2000) programming language. Forward and reverse read pairs in interleaved FASTQ format were assembled using FLASH (Magoč and Salzberg, 2011) version 1.2.11 specifying the ‘–interleaved-input’ and ‘–flash-max-overlap 100’ command line flags. Using blastn from the NCBI blast package (Camacho et al., 2009) version 2.10.1+, the assembled reads were aligned as query sequences against a database containing the single target DNA sequence (in this case, TAP-1, KY497396.1). Alignment parameters were highly permissive with following blastn command line flags: ‘-gapopen 1 -gapextend 1 -xdrop_gap 90 -xdrop_gap_final 300 -max_target_seqs 1’. For ease of parsing, custom output of comma-separated values with was specified as follows: ‘-outfmt 10 qseqid qstart qseq qend sseqid sstart sseq send pident nident mismatch gapopen gaps qlen’. In order to allow grouping of identical CRISPR edits regardless of the sequence completeness, query sequences in the blastn output were adjusted to the same orientation as the reference, and the complete 5′ and 3′ flanking sequences derived from the reference sequence were appended to the query. This allowed the adjusted query sequences to be used as hash keys for tabulation of statistics of grouped identical CRISPR-edited sequences. For each query sequence, BLAST alignment start and end coordinates of the query (*qstart*, *qend*) and subject (*qstart*, *qend*) were used to calculate indel size (|*qend – qstart| - |send – sstart|*) and frameshifts (*(|qend – qstart| - |send – sstart|) % 3*). Grouping by adjusted query sequence, these and additional statistics including the number of times that an adjusted query sequence is observed, and the corresponding percentage out of all aligned sequences. These and other statistics were saved to output files. The overall tabulated frameshift and indel counts are also output and plotted using the R language’s base barplot() and pie() functions (R Core Team, 2017).

#### Antibodies and flow cytometry

Flow cytometric analyses were performed using HLA-ABC (W6/32) APC (1:100; Biolegend) (Parham et al., 1979) and LIVE/DEAD violet viability dye (1:1000; Life Technologies). Cell surface staining of HLA expression was performed on cells grown in 96-well plates in 200 μL volume. Cells were stained with antibody and viability dye in PBS + 2% FBS for 20 min at 4°C and fixed in 4% paraformaldehyde, prior to flow cytometric analysis using a BD LSR II (BD Biosciences). Flow cytometric data were analyzed using FlowJo software (v10.1r5; Treestar).

#### Peptide synthesis reagents

Fmoc-protected amino acids and synthesis resin, 2-Chlorotrityl chloride were purchased from Akaal Organics (Long Beach, CA). Dimethylformamide (DMF), N-methyl pyrrolidone (NMP), Acetonitrile and Methyl-tert. Butyl Ether (MTBE) were purchased from Fisher Bioreagents (Fair Lawn, NJ). 2-(6-Chloro-1-H-benzotriazole-1-yl)-1,1,3,3-tetramethylaminium hexafluorophosphate (HCTU) was purchased from AAPPTEC (Louisville, KY). Piperidine and Dichloromethane (DCM) were from EMD-Millipore (Billerica, MA). Diisopropylethylamine (DIEA), N-Methyl-morpholine (NMM), Triisoprpopyl-silane, 3,6-dioxa-1,8-octanedithiol (DODT) and trifluoroacetic acid (TFA) were purchased from Sigma–Aldrich.

#### Peptide synthesis and analysis

Peptides were synthesized on an automated robotic peptide synthesizer (AAPPTEC, Model 396 Omega) by using Fmoc solid-phase chemistry (Behrendt et al., 2016) on 2-chlorotrityl chloride resin (Barlos et al., 1991). The C-terminal amino acids were loaded using the respective Fmoc-Amino Acids in the presence of DIEA. Unreacted sites on the resin were blocked using methanol, DIEA and DCM (15:5:80 v/v). Subsequent amino acids were coupled using optimized (to generate peptides containing more than 90% of the desired full-length peptides) cycles consisting of Fmoc removal (deprotection) with 25% Piperidine in NMP followed by coupling of Fmoc-AAs using HCTU/NMM activation. Each deprotection or coupling was followed by several washes of the resin with DMF to remove excess reagents. After the peptides were assembled and the final Fmoc group removed, peptide resin was then washed with dimethylformamide, dichloromethane, and methanol three times each and air-dried. Peptides were cleaved from the solid support and deprotected using odor free cocktail (TFA/triisopropyl silane/water/DODT; 94/2.5/2.5/1.0 v/v) for 2.5h at room temperature (Teixeira et al., 2002). Peptides were precipitated using cold methyl tertiary butyl ether (MTBE). The precipitate was washed 2 times in MTBE, dissolved in a solvent (0.1% trifluoroacetic acid in 30%Acetonitrile/70%water) followed by freeze drying. Peptides were characterized by Ultra Performance Liquid Chromatography (UPLC) and Matrix Assisted Laser Desorption/Ionization Mass Spectrometry (MALDI-MS). All peptides were dissolved initially in 100% DMSO at a concentration of 40 mM, prior to dilution at the appropriate concentration in RPMI-1640 medium.

#### HLA class I-peptide concentration-based stability assay

For concentration-based HLA class I-peptide stability binding assays, 5x10^4^ TAP-deficient mono-allelic HLA class I expressing 721.221 cells were incubated with peptides in concentrations ranging from 0.1 to 100 μM, and 3 μg/mL of β2 m (Sigma-Aldrich, St. Louis, MO, USA; Sino Biological, Wayne, PA, USA), in RPMI-1640 medium overnight at 26°C/5% CO2 for 18 hours. Controls without peptide, but the corresponding concentration of DMSO, were performed in parallel. Following overnight incubation, cells were incubated at 37°C/5% CO2 prior to staining for viability and HLA class I surface expression with HLA-ABC APC antibody (1:100), and subsequent analysis by flow cytometry.

#### Brefeldin A HLA-class I-peptide time-based assay

For time-based HLA class I-peptide stability binding assays, 5x10^4^ TAP-deficient mono-allelic HLA class I expressing 721.221 cells were incubated with peptides at a concentration of 10 μM and 3 μg/mL of β2 m (Sigma-Aldrich, St. Louis, MO, USA; Sino Biological, Wayne, PA, USA), in RPMI-1640 medium overnight at 26°C/5% CO2 for 18 hours. Following overnight incubation, cells were washed twice with 1X PBS and resuspended in RPMI-1640 medium containing 5 μg/mL of Brefeldin A (BFA) (Biolegend). To determine the baseline values at *t* = 0, cells were stained for viability and HLA class I surface expression with HLA-ABC APC antibody (1:100) following overnight incubation. At indicated time points, the cells were stained and evaluated by flow cytometry.

#### HLA monomer expression and purification

Expression, refold and purification of the soluble constructs of the pMHCs were performed as previously described (Pierce et al., 2014). Briefly, the HLA-A^∗^02, HLA-A^∗^03 and HLA-B^∗^57 heavy chains, and β2-microglobulin were expressed in *Escherichia coli* and purified as inclusion bodies (IBs). The IBs were solubilized in 8M Urea. Target peptides were provided by the MGH Peptide Core. For HLA-A^∗^02 and HLA-A^∗^03 refold, the heavy chain, β2 m and the peptide were added to a refolding buffer containing 100 mM Tris at pH 8.0, 2 mM EDTA, 400 mM L-arginine, 0.5 mM oxidized glutathione, 5 mM reduced glutathione, and 0.2 mM PMSF in a molar ratio of 1:3:10 respectively. The reaction mix was first incubated at 4°C for 24 hours and then dialyzed against 10 mM Tris for the next 60 hours at room temperature. The pMHC complexes were purified using Ni column followed by size exclusion chromatography. In case of HLA-B^∗^57 refold, the heavy chain, β2 m and the peptide were added to a refolding buffer containing 100 mM Tris at pH 8.3, 2 mM EDTA, 400 mM L-arginine, 4 M Urea, 1 mM oxidized glutathione, 1.5 mM reduced glutathione, and 0.2 mM PMSF in a molar ratio of 1:3:5 respectively. The reaction mix was first incubated at 4°C for 24 hours and followed by dialysis against 10 mM Tris for the next 60 hours at 4°C. The pMHC complexes were purified using DEAE column followed by size exclusion chromatography.

#### Differential scanning fluorimetry

Differential scanning fluorimetry was performed using a Bio-RAD CFX96 real time PCR system as previously described. Briefly, the excitation and emission wavelengths were set to 587 and 607 nm respectively, and the fluorescence intensity was measured after every 1°C rise in temperature starting from 20°C and going up to 95°C. Each reaction mix contained 19.8 μL of 2 μM pMHC (buffer: 10mM HEPES at pH 7.4, 150 mM NaCl, 3 mM EDTA, and 0.005% surfactant P20) and 0.2 μL of 1000X SYPRO orange dye. Apparent *T*_m_ values were calculated by identifying the point at which the melting transition was 50% complete.

### Quantification and statistical analysis

The generation of dot plots, nonparametric statistical analysis, correction for multiple comparisons and non-parametric correlations (Spearman) were performed using the statistical programs in Graphpad Prism version 8.0. Differences between groups were evaluated using the non-parametric Mann Whitney U t test and Kruskal-Wallis test with Dunn’s post hoc analyses for correction of multiple comparisons, as indicated. Paired analyses were performed using the non-parametric Wilcoxon matched-pairs signed rank test. All statistical details and p values can be found in figure legends.

## Supplemental information

Document S1. Figures S1–S5

Table S1. List of HIV epitopes tested by HLA class I-peptide stability assay, related to Figures 2 and 5

Document S2. Article plus supplemental information
