# Supplementary material for: HLA class-I-peptide stability mediates CD8+ T cell immunodominance hierarchies and facilitates HLA-associated immune control of HIV
Source: Cell Rep. 2021 Jul 13;36(2):109378. doi: 10.1016/j.celrep.2021.109378 (PMC8293625; doi:10.1016/j.celrep.2021.109378)
Supplement: Document S1. Figures S1–S5 [file mmc1.pdf]

Cell Reports, Volume *36*

Supplemental information

HLA class-I-peptide stability mediates CD8^{+} T cell immunodominance hierarchies and facilitates HLA-associated immune control of HIV

Clarety Kaseke, Ryan J. Park, Nishant K. Singh, Dylan Koundakjian, Arman Bashirova, Wilfredo F. Garcia Beltran, Overbeck C. Takou Mbah, Jiaqi Ma, Fernando Senjobe, Jonathan M. Urbach, Anusha Nathan, Elizabeth J. Rossin, Rhoda Tano-Menka, Ashok Khatri, Alicja Piechocka-Trocha, Michael T. Waring, Michael E. Birnbaum, Brian M. Baker, Mary Carrington, Bruce D. Walker, and Gaurav D. Gaiha

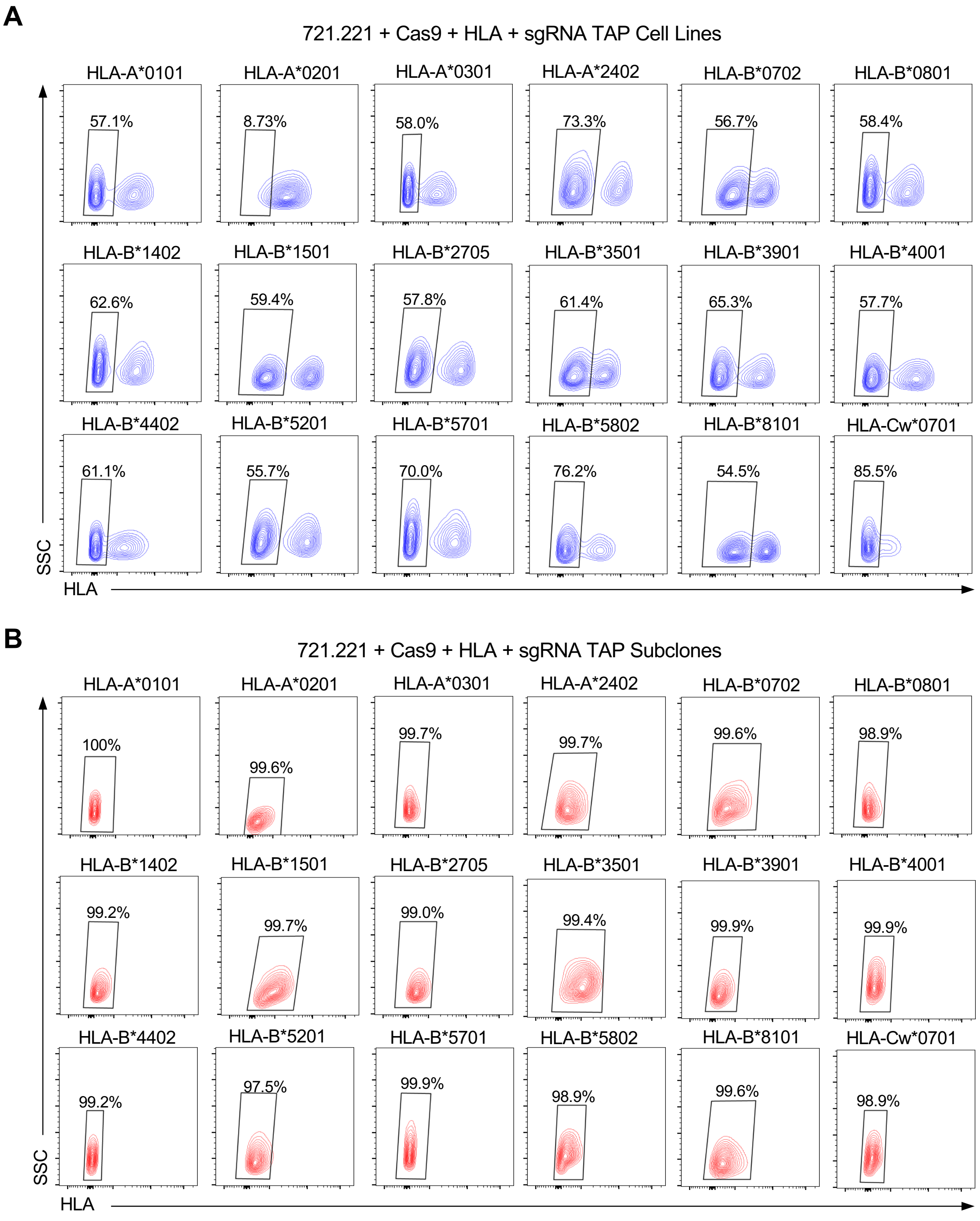

Figure S1. HLA class I surface expression of CRISPR/Cas9-edited TAP-deficient cell lines and subclones, Related to Figure 1. (A) Flow cytometry analysis of surface HLA expression, as determined by staining with pan-HLA antibody W6/32, for all 18 mono-allelic HLA class I-expressing 721.221 cells following transduction with sgRNA TAP and selection with G418. Cells with negative HLA class I surface expression ranged from 8.7-85.5%. (B) Flow cytometry analysis of surface HLA expression for all 18 mono-allelic HLA class I-expressing 721.221 cells following limiting dilution subcloning. Cells with negative HLA class I surface expression ranged from 97.5-100%.

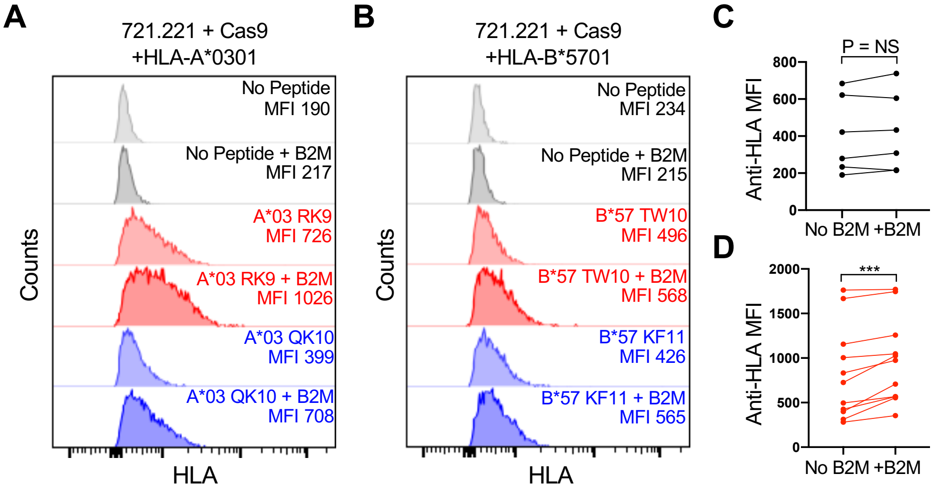

Figure S2. Addition of soluble β2m enhances peptide-mediated stabilization of HLA class I molecules, Related to Figure 1. (A) Representative surface stabilization of HLA-A*0301 molecules with no peptide, RK9 peptide (Gag p17 20-28) or QK10 peptide (Nef 73-82) in the presence or absence of β2m (3 µg/mL) or (B) HLA-B*5701 molecules with no peptide, TW10 peptide (Gag p24 108-117) or KF11 peptide (Gag p24 30-40) in the presence or absence of β2m (3 µg/mL). (C, D) Comparison of surface HLA class I expression in the presence or absence of β2m for no peptide and peptide conditions, respectively. Statistical comparisons were made using Wilcoxon matched-pairs signed rank test. Calculated P values were as follows: NS = non-significant, *P < 0.05; **P < 0.01; ***P < 0.001; ****P < 0.0001.

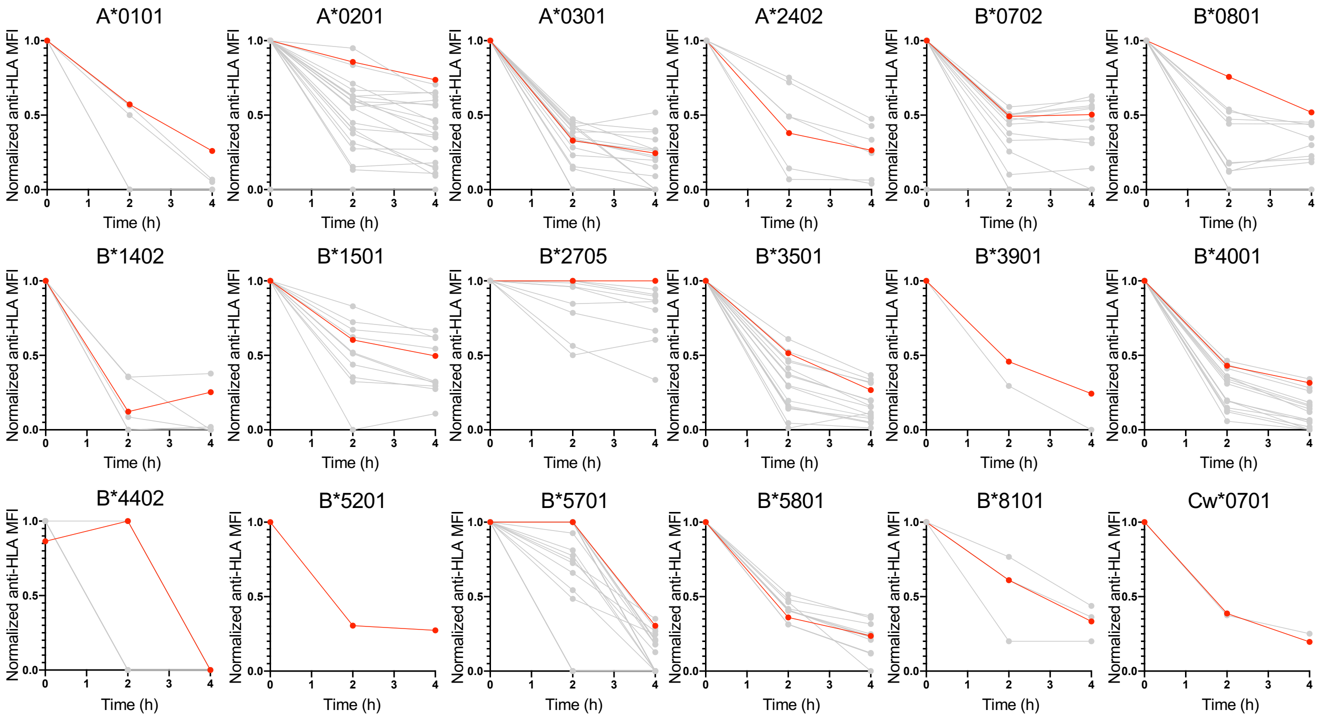

Figure S3. Time-based HLA class I-peptide stabilization of HIV epitopes, Related to Figure 2. Stabilization of HLA class I molecules with 186 known optimal HIV CD8^{+} T cell epitopes (10µM) in the presence of BFA (5µg/mL) across 18 TAP-deficient mono-allelic HLA class I-expressing cell lines. The *y* axis depicts the anti-HLA MFI normalized to the highest value for each HLA class I allele (0-1). Known immunodominant HIV epitopes based on frequency of CD8^{+} T cell targeting are indicated in red.

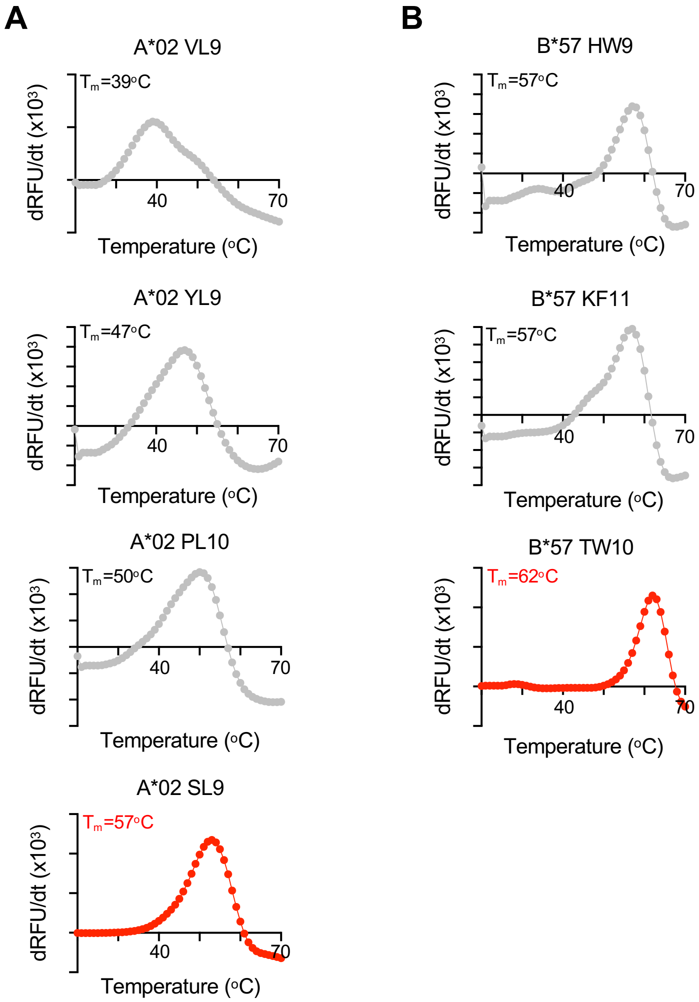

Figure S4. Thermal denaturation of soluble HLA-A*02 and HLA-B*5701 monomers in complex with immunodominant or subdominant HIV epitopes, Related to Figure 3. (A, B) Representative thermal denaturation of HLA-A*0201 and HLA-B*5701 peptide monomers for indicated HIV epitopes, respectively. The *x* axis depicts temperature (20-70°C). The *y* axis depicts the derivative of the temperature versus fluorescence (-dRFU/dT). The thermal stability (Tm) is indicated for each HLA-peptide complex. Each thermal denaturation was performed twice as technical triplicates.

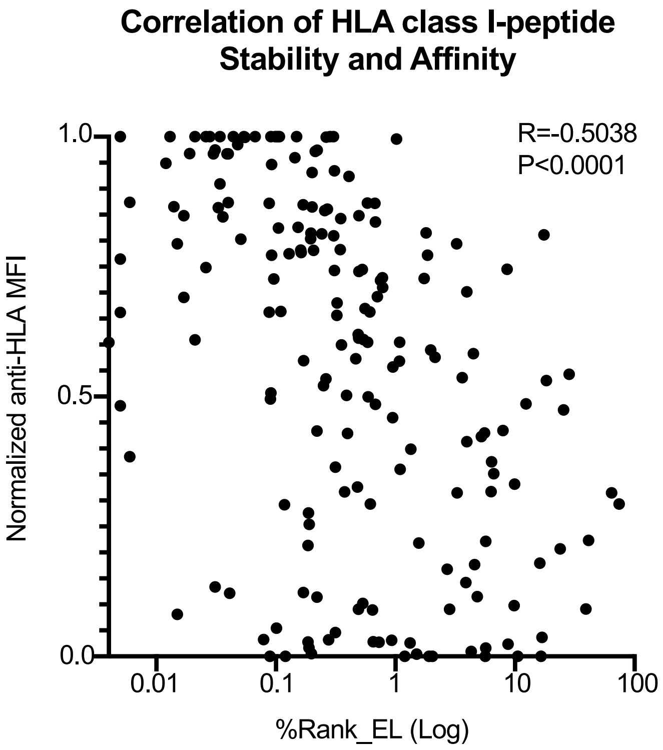

Figure S5. Correlation of relative HLA class I-peptide stability to predicted HLA affinity for all HIV epitopes, Related to Figure 4. Scatter plot of NetMHCPan 4.1 affinity values (log 10, x axis) with relative anti-HLA MFI values (y axis) for each HIV epitope evaluated in the study. Correlation was calculated by Spearman's rank correlation coefficient.
